# Comparing Highly Efficacious Antimalarial Drugs

**DOI:** 10.1371/journal.pmed.0050228

**Published:** 2008-11-18

**Authors:** Colin J Sutherland

## Abstract

Colin Sutherland discusses a Policy Forum article that explores the design and standardization of pre-licensure phase III antimalarial treatment trials.

It is almost ten years since a clarion call was sounded to malaria researchers, funding agencies, governments, and international organisations to help avert “a malaria disaster” [1]. At that time, infrastructure in malaria control programmes across Africa was deteriorating. This deterioration was exacerbated by an alarmingly high prevalence of parasites resistant to the two affordable treatments being used across the continent: chloroquine and sulphadoxine-pyrimethamine. The plea was to speed up the introduction of combination therapies in which one of the component drugs was from a highly effective antimalarial class, the artemisinins, derived from the Chinese herbal remedy *qinghaosu*. Subsequent investment in research and clinical trials, and new drug procurement arrangements through the Global Fund to Fight AIDS, Tuberculosis and Malaria, have achieved much to be proud of. Many African countries have now implemented new malaria treatment policies centred around artemisinin combination therapy (ACT) as the front-line treatment for malaria.

## Testing New Combinations Against Current Regimens

There is now an urgent need to bring new combination therapies into the malaria drug development pipeline, to provide endemic country governments with alternative regimens suited to malaria transmission in their setting, and to minimise the global impact of resistance to ACTs, should it arise. However, in order to be granted licensure, such new combinations must be tested against current ACT-based regimens of high efficacy. Therefore the previously established phase III clinical trial designs, which test for *superiority* of the investigational product against a failing drug such as chloroquine or sulphadoxine-pyrimethamine, can no longer apply. There are now important questions about the design and standardisation of such pre-licensure phase III antimalarial treatment trials.

**Linked Policy Forum**

This Perspective discusses the following new article published in *PLoS Medicine*:

Borrmann S, Peto T, Snow RW, Gutteridge W, White NJ (2008) Revisiting the design of phase III clinical trials of antimalarial drugs for uncomplicated Plasmodium falciparum malaria. PLoS Med 5(11): e227. doi:10.1371/journal.pmed.0050227

Steffen Borrmann and colleagues discuss appropriate endpoints and their measurement during phase III trials of new antimalarial drugs.

In a new Policy Forum on this topic, Steffen Borrmann and colleagues make important recommendations for such trials [2]. In particular, they advocate the use of a non-inferiority study design when deciding if a new regimen has an acceptable level of performance. In such a design, a new therapy is deemed acceptable for further investigation or licensure if it performs “at least as well as” a current therapy of good efficacy. The precise meaning of this phrase must be widely agreed before this approach can be adopted, and before future standardisation of such studies can be established. Borrmann and colleagues throw this debate open, but themselves advocate a “delta margin” (see Glossary) of 5%, with a fixed benchmark of at least 90% cure rate. Thus the efficacy of any new combination would be required to reach 95% of the efficacy of the established regimen, *and* cure at least 90% of treated patients in a phase III study: violation of either criterion would mean the new therapy was not deemed of adequate efficacy.

## The Question of Efficacy Endpoints

However, we encounter dangerous waters when the question of efficacy endpoints arises—what are the appropriate measures of antimalarial efficacy when testing for non-inferiority with an established regimen in phase III studies? A feature of falciparum malaria efficacy studies over the last decade has been the use of so-called “PCR correction” (see Glossary) to distinguish between treatment failure caused by recrudescent parasites (i.e., those that are identical to pre-treatment parasites by genetic fingerprinting) and treatment failure caused by genetically distinct parasites that have emerged from the liver after treatment. Borrmann and colleagues skilfully navigate these waters, and finally recommend that the efficacy endpoint for phase III trials should be the absence of recrudescent parasites over 28 days of follow-up, verified by PCR correction [2].

An alternative view is that uncorrected estimates of antimalarial efficacy, combining therapeutic and prophylactic effects in a single measure, should be given primacy. The main justification for this unfashionable opinion is 2-fold. Firstly, there is no evidence of a clinically meaningful difference between recrudescent and newly emergent infections [3]. Secondly, PCR-corrected estimates of efficacy cannot be directly compared across studies. This is because the gap between corrected and uncorrected estimates will differ between trial sites as transmission intensity, levels of acquired immunity, prevalence of parasite resistance to ACT partner drugs (e.g., amodiaquine, lumefantrine), and the genetic complexity of the parasite population vary. These factors will influence the “PCR-corrected” estimate of efficacy, but only one (prevalence of resistance) is truly a component of “drug efficacy”. Thus PCR-corrected estimates of efficacy for a particular regimen in different sites should only be compared by normalising against a well-characterised comparator drug tested in both sites, using harmonised protocols, and this comparison should be on a population level, and not by re-classification of individual treatment failures [4].

**Glossary**

**Delta margin:** In the context of non-inferiority clinical trials, the delta margin sets the efficacy benchmark below which the test drug fails to be “non-inferior” to the comparator drug. This benchmark is simply the measured efficacy of the comparator drug minus the delta margin; for a comparator with measured efficacy of, say, 96%, and a delta margin of 5%, the test drug must reach a measured efficacy of 91.2%.

**PCR correction:** Use of genetic fingerprinting techniques, usually based on the polymorphic antigen loci *pfmsp1* and *pfmsp2*, to determine whether P. falciparum parasites recurring in a patient's peripheral blood after antimalarial treatment are genetically identical to, or different from, the parasites present prior to treatment. If identical to pre-treatment parasites, the recurrent infection is considered to be a recrudescence; if different it is considered to be newly emergent from the liver and not a treatment failure, *sensu stricto*. There is disagreement in the literature as to the validity of this approach for correcting estimates of drug efficacy.

## Alternative Ways To Monitor Drug Performance

There are other ways of monitoring the performance of antimalarial drugs not considered by Borrmann and colleagues. Transmission endpoints, such as gametocyte carriage or the infectivity of treated individuals to Anopheles mosquitoes, provide early signals of developing P. falciparum resistance to antimalarial drugs, and could be more widely applied to comparative efficacy studies [5,6]. Efficacious drugs may also differ in the speed with which pre-treatment parasite densities are reduced, and a recent trial has used the parasite clearance time, monitored by repeat peripheral blood sampling over days 1 to 3 post-treatment, to distinguish between regimens with good 14-day efficacy [7]. This approach, simplified to reduce the number of samples required, should also be used to monitor ACT efficacy for signs of increasing parasite clearance time as these valuable regimens are deployed across the globe.

## Abbreviations

ACT: artemisinin combination therapy
